# Two dominant loci determine resistance to Phomopsis cane lesions in F_1_ families of hybrid grapevines

**DOI:** 10.1007/s00122-018-3070-1

**Published:** 2018-02-21

**Authors:** Paola Barba, Jacquelyn Lillis, R. Stephen Luce, Renaud Travadon, Michael Osier, Kendra Baumgartner, Wayne F. Wilcox, Bruce I. Reisch, Lance Cadle-Davidson

**Affiliations:** 1 Plant Breeding and Genetics Section, School of Integrative Plant Science, Cornell University, Ithaca, NY 14853 USA; 2 USDA-ARS Grape Genetics Research Unit, Geneva, NY 14456 USA; 3 Horticulture Section, School of Integrative Plant Science, New York State Agricultural Experiment Station, Cornell University, Geneva, NY 14456 USA; 4 Department of Plant Pathology, University of California, Davis, CA 95616 USA; 5 Thomas H. Gosnell School of Life Sciences, Rochester Institute of Technology, Rochester, NY 14623 USA; 6 USDA-Agricultural Research Service, Crops Pathology and Genetics Research Unit, Davis, CA 95616 USA; 7 Plant Pathology Section, School of Integrative Plant Science, New York State Agricultural Experiment Station, Cornell University, Geneva, NY 14456 USA; 8 Present Address: Instituto de Investigaciones Agropecuarias, INIA La Platina, Santa Rosa, 11610 Santiago, Chile

## Abstract

**Key message:**

Rapid characterization of novel NB-LRR-associated resistance to Phomopsis cane spot on grapevine using high-throughput sampling and low-coverage sequencing for genotyping, locus mapping and transcriptome analysis provides insights into genetic resistance to a hemibiotrophic fungus.

**Abstract:**

Phomopsis cane and leaf spot, caused by the hemibiotrophic fungus *Diaporthe ampelina* (syn = *Phomopsis viticola*), reduces the productivity in grapevines. Host resistance was studied on three F_1_ families derived from crosses involving resistant genotypes ‘Horizon’, Illinois 547-1, *Vitis cinerea* B9 and *V. vinifera* ‘Chardonnay’. All families had progeny with extremely susceptible phenotypes, developing lesions on both dormant canes and maturing fruit clusters. Segregation of symptoms was observed under natural levels of inoculum in the field, while phenotypes on green shoots were confirmed under controlled inoculations in greenhouse. High-density genetic maps were used to localize novel qualitative resistance loci named *Rda*1 and *Rda*2 from *V. cinerea* B9 and ‘Horizon’, respectively. Co-linearity between reference genetic and physical maps allowed localization of *Rda*2 locus between 1.5 and 2.4 Mbp on chromosome 7, and *Rda*1 locus between 19.3 and 19.6 Mbp of chromosome 15, which spans a cluster of five NB-LRR genes. Further dissection of this locus was obtained by QTL mapping of gene expression values 14 h after inoculation across a subset of the ‘Chardonnay’ × *V. cinerea* B9 progeny. This provided evidence for the association between transcript levels of two of these NB-LRR genes with *Rda*1, with increased NB-LRR expression among susceptible progeny. In resistant parent *V. cinerea* B9, inoculation with *D. ampelina* was characterized by up-regulation of SA-associated genes and down-regulation of ethylene pathways, suggesting an R-gene-mediated response. With dominant effects associated with disease-free berries and minimal symptoms on canes, *Rda*1 and *Rda*2 are promising loci for grapevine genetic improvement.

**Electronic supplementary material:**

The online version of this article (10.1007/s00122-018-3070-1) contains supplementary material, which is available to authorized users.

## Introduction

Agricultural producers are facing increasing pressure to reduce the use of fungicides, for which deployment of cultivars with disease resistance is one viable solution. Accordingly, several sources of resistance to major crop diseases have been identified and introgressed. For example, in grapes the two most important foliar diseases, powdery mildew and downy mildew, can be suppressed by resistance genes identified from wild sources (Blasi et al. 2011; Feechan et al. 2013; Mahanil et al. 2012; Ramming et al. 2011). Once genetic control of major diseases and the subsequent reduction in fungicide applications is achieved, other problems may emerge, namely pathogens that were secondary targets of routine fungicide applications. This phenomenon has been observed in powdery and downy mildew-resistant vineyards, where the incidence of grapevine black rot increased (Molitor and Beyer 2014; Rex et al. 2014).

Phomopsis cane and leaf spot of grapevine (“Phomopsis”) is caused by *Diaporthe ampelina* (Ascomycota, Diaporthales; syn. = *Phomopsis viticola*,) (Gomes et al. 2013; Wilcox et al. 2015). Most *Diaporthe* species are considered hemibiotrophic (Udayanga et al. 2011), with an initial biotrophic phase of plant tissue colonization before the necrotrophic phase, when lesions or cankers develop. In grapevine, leaf and cane infections by *D. ampelina* are initiated by rain-splashed conidia released from pycnidia present on previously infected tissues. Dispersed conidia adhere to the plant tissues and under suitable conditions, germinate and penetrate tissues through stomatal pores or wounds (Pine 1959). Leaf and cane infections require a minimum of 7 h wetness duration at optimum temperatures of 16–20 °C (Erincik et al. 2003). In plant tissues, the mycelium germinating from conidia invades the cortical parenchyma and forms pseudo-parenchymatous mats among host cells. Host cells become necrotic and shoot lesions and leaf spots usually appear 3–4 weeks after infection (Wilcox et al. 2015). New pycnidia form on these necrotic lesions, providing inoculum for new infections. Lesions remain after lignification in dormant canes, resulting in shoot breakage. On clusters, Phomopsis can cause lesions on the rachis, resulting in loss of up to 30% of yields (Anco et al. 2012).

In Mediterranean climates (e.g., California), foliar symptoms are less common, but *D. ampelina* and other *Diaporthe* species are instead more frequently associated with the formation of wood cankers (Lawrence et al. 2015), Phomopsis dieback being part of the grapevine trunk-disease complex (Úrbez-Torres et al. 2013). In controlled experiments, grapevine cultivars responded differently to wood infection by *D. ampelina* (Travadon et al. 2013), suggesting a genetic component in the plant–pathogen interaction. To date, the genetic and molecular bases of Phomopsis resistance in grapevines have not been reported.

In the plant immune response, pathogen-associated molecular patterns (PAMPs) are recognized by pathogen recognition receptors, triggering a defense response known as PAMP-triggered immunity (PTI). The pathogen can escape this defense response by deploying effectors. In response, plants utilize a surveillance mechanism mediated by R-genes coding for proteins characterized by a nucleotide-binding site leucine-rich repeats (NB-LRR). Upon recognition of pathogen effectors, a cascade of reactions leads to a hypersensitive response [effector-triggered immunity (ETI)] (Jones and Dangl 2006). This type of response is associated with the production of reactive oxygen molecules and localized cell death, mediating the resistance to biotrophic and hemibiotrophic fungi (Greenberg and Yao 2004; Morel and Dangl 1997). Defenses against biotrophic pathogens are also regulated by a salicylic acid (SA)-dependent pathway, which plays a role in both local defense reactions and induction of systemic acquired resistance (Durner et al. 1997). In contrast, defenses against necrotrophic pathogens are regulated by induction of jasmonic acid (JA) and ethylene signaling (Glazebrook 2005). In the plant defense response, there is an antagonistic cross talk between SA and both ethylene and JA pathways, as well as SA and auxin signaling pathways (Kazan and Manners 2009).

R-genes are often major dominant genes that provide complete or qualitative disease resistance, becoming interesting targets for introgression in breeding programs. Over time, plant pathogens can modify their effectors, avoiding recognition, and thus resistance mediated by R-genes can be overcome in new cultivars after their deployment (Jones and Dangl 2006; Peressotti et al. 2010). Stacking of several loci has been proposed as a mechanism to prolong the durability of R-genes, but the selection of multiple loci that generate the same phenotype requires the use of molecular markers through marker-assisted selection (MAS).

In this paper, we report our study into the genetics of Phomopsis resistance of canes and clusters in three hybrid grapevine families. First, we quantified the segregation of cane and cluster symptoms in families derived from interspecific hybrids ‘Horizon’ and Illinois 547-1, *V. vinifera* ‘Chardonnay’, and *V. cinerea* B9. We used high-density genetic maps to study the association between phenotype and molecular markers, which allowed further identification of two novel major resistance loci. Candidate genes were further dissected through gene expression analysis.

## Materials and methods

### Plant material

Three related full sib, F_1_ families were derived from the cross of four parental genotypes: *V. vinifera* ‘Chardonnay’ clone 95, *V. cinerea* B9, Illinois 547-1 (*V. rupestris* B38 × *V. cinerea* B9) and the complex hybrid ‘Horizon’ (‘Seyval’ × ‘Schuyler’, whose pedigree includes *V. vinifera, V. labrusca, V. aestivalis* and *V. rupestris* (Reisch et al. 1982). The ‘Horizon’ × Illinois 547-1 family (366 vines) resulted from crosses made in 1988 (Dalbó et al. 2000) and 1996. Families ‘Horizon’ × *V. cinerea* B9 (162 vines) and ‘Chardonnay’ × *V. cinerea* B9 (148 vines) resulted from crosses made in 2009. For all families, in the year following cross-hybridization, seeds were stratified prior to germination, and seedlings were grown in an irrigated field nursery. Two years after cross-hybridization, vines were transplanted to a permanent vineyard in Geneva, New York. Single vines per genotype were planted 1.2 m apart. A control block was included in each row, with the following genotypes: *V. vinifera* ‘Chardonnay’ (susceptible to powdery and downy mildew), *V*. hybrid ‘Chancellor’ (Seibel 5163 × Seibel 880) (susceptible to powdery and downy mildew), *V. rupestris* B38 (resistant to powdery and downy mildew, *V*. hybrid ‘Horizon’ (‘Seyval’ × ‘Schuyler’) (intermediate resistance to powdery and downy mildew) and the breeding selection NY88.0514.04 (resistant to powdery and downy mildew). Powdery mildew-susceptible control ‘Chardonnay’ was planted after every 15 seedling vines. Parental lines *V. cinerea* B9 and Ill. 547-1 are also classified as resistant to powdery and downy mildew.

Fungicide applications were reduced to the minimum necessary to maintain plant viability. *N*-trichloromethylthio-4-cyclohexene-1,2-dicarboximide (Captan 80WDG) was applied at recommended rates at the following phenological stages [identified according to the modified Eichhorn–Lorenz scale (Coombe 1995)] during 2011 through 2013: stage 12 (1.68 kg/ha, late May), stage 17–18 (2.24 kg/ha, early June), stage 26 (2.80 kg/ha, mid-June), stage 29 (2.80 kg/ha, late June) and stage 31 (2.80 kg/ha, mid-July). Potassium phosphite (ProPhyt, Helena Chemical Company, Collierville, TN, USA) was applied for control of downy mildew at a rate of 2.35 kg/ha of phosphorous acid equivalent at stages 32 and 34 (early and mid-August, respectively) in 2011 and 2012.

After assessment of symptoms, field vines were vegetatively propagated for further experiments. First, *V. cinerea* B9 vines were propagated in 2010 as described previously (Barba et al. 2015): briefly, dormant cuttings were taken from the vineyard, stored at 4 °C and potted in a 3:1 mixture of perlite: soil with bottom heat at 26 °C until sufficient rooting took place. Vines were then grown in a greenhouse under a 16 h photoperiod at 27–30 °C, pruned and stored at 4 °C for dormancy. In 2011, potted vines were grown in a greenhouse as described above and pruned to assure uniform vegetative growth when needed. Secondly, dormant cuttings from resistant and susceptible plants were sent to California to confirm phenotypes under controlled conditions (i.e., symptoms were due to *D. ampelina* alone). From cross ‘Chardonnay’ × *V. cinerea* B9, four resistant progenies (454064, 455035, 454053, 454058) and six susceptible progenies (454066, 454071, 455072, 455082, 454045, 454077) were propagated. Twelve replicates per genotype were established in the greenhouse at the University of California Experiment Station in Davis as a source of green cuttings for the following experiments. Dormant cuttings taken from the New York vineyard were surface sterilized in 1% sodium hypochlorite for 15 min, soaked in water overnight and then callused in boxes filled with perlite and vermiculite (1:1, vol/vol) for 21 days at 30 °C and 85% relative humidity. After callusing, cuttings were planted in sleeves in a 1:1 mixture of perlite and vermiculite and then returned to 30 °C at 85% relative humidity for 14 days to further encourage callusing. Plants were afterward placed under intermittent water mist (5 s every 2 min during daylight) at 28 °C for 7 days in the greenhouse, at which point leaves emerged and plants were then transferred to the greenhouse and potted in UC mix (Baker 1957). After 5 months [natural sunlight photoperiod, 25 ± 1 °C (day) and 18 ± 3 °C (night)], there was sufficient shoot growth for propagation of plants from green cuttings for inoculation experiments.

### Field disease evaluation

**Fig. 1:**
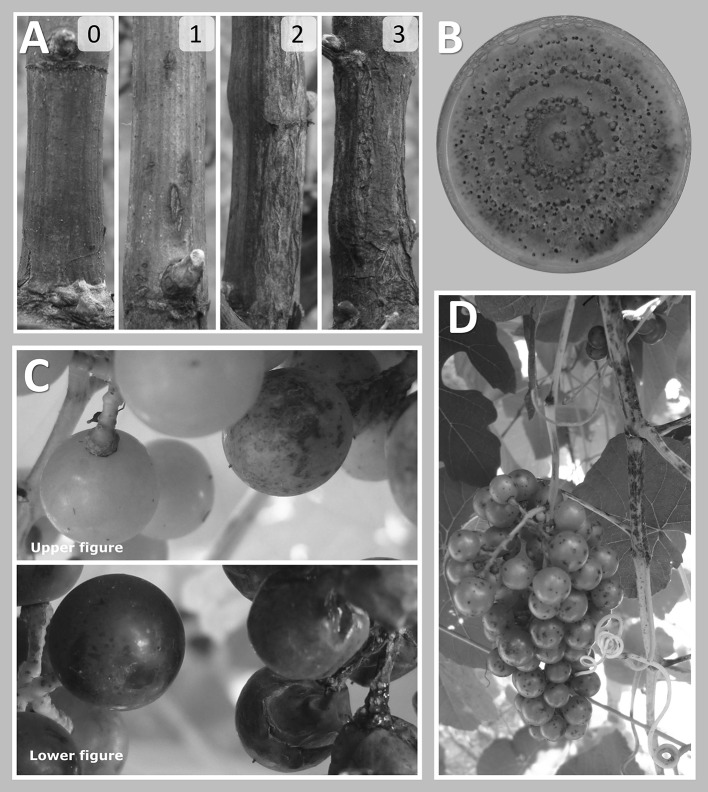
Symptoms and *Diaporthe ampelina* isolation. **a** Phomopsis cane symptoms were scored on dormant canes using the following scale: (0) no Phomopsis symptoms observed; (1) light infection, small number of discrete lesions; (2) moderate infection, lesions coalescing, widespread; and (3) severe infection, lesions blackened, internode tissue corky and misshapen. **b** *D. ampelina* culture isolated from symptomatic canes (score 3), growing on potato dextrose agar. **c** Progression of symptoms on resistant (left) and susceptible (right) full siblings growing side by side in the vineyard on August 21 (upper) and September 10 (lower), 2013. **d** Phomopsis symptoms on green shoots and unripe berries

Field vines were subject to natural inoculation. Phomopsis symptoms were evaluated on dormant canes each autumn from 2011 through 2013 using the following disease severity scale: (0) no symptoms; (1) light infection, few discrete circular lesions; (2) moderate infection, widespread coalescing circular lesions; (3) severe infection, widespread coalescing misshapen lesions with blackened surface and corky texture (Fig. 1a). Symptoms on clusters were scored as present or absent before veraison in 2013 and 2014. As male vines did not set fruit, the number of samples was reduced to 65 observations in the ‘Horizon’ × *V. cinerea* B9 family and 58 observations in the ‘Chardonnay’ × *V. cinerea* B9 family. No cluster observations were made in the ‘Horizon’ × Illinois 547-1 family.

### *Diaporthe ampelina* isolation from the field

Canes from diseased vines located in Geneva, NY, were collected during the spring of 2013 and incubated in a clean, sealed plastic box with wet paper towels to provide humidity. *Diaporthe ampelina* conidia were collected from oozing lesions and plated on potato dextrose agar (PDA, Difco Laboratories, Detroit, MI, USA), and emerging colonies were subcultured onto fresh PDA plates. Cultures were maintained at room temperature under fluorescent light and transferred to fresh PDA every 3–4 weeks. For RNA-Seq experiments, controlled inoculations were made using a solution of conidia obtained by flooding pycnidia-bearing colonies on PDA plates with sterile distilled water; after approximately 5 min, the resulting spore suspension was decanted and diluted in sterile water plus Tween 20 (10 µl/l) to a final concentration of 10^7^ conidia/ml.

### Confirmation of phenotypes after *D. ampelina* inoculation

To confirm the phenotypes observed under NY field conditions were due to *D. ampelina* alone, the following experiment was performed: Two-bud green cuttings were taken from green shoots of California greenhouse stock plants in October 2015, December 2015 and February 2016, corresponding to three independent, replicated experiments. Cuttings were rooted in perlite in the greenhouse [natural sunlight photoperiod, 25 ± 1 °C (day), 18 ± 3 °C (night)] with intermittent water mist (5 s every 2 min during daylight). Roots formed after 2 weeks, at which point cuttings were transplanted into a potting mix of peat, sand and perlite (1:1:1, v/v/v) in plastic trays (58 × 40 × 22.5 cm; XL High Dome Propagator, Garland Products, England). For each of the three experiments, a total of 24 inoculated plants per genotype [parental vines *V. cinerea* B9 and ‘Chardonnay’, four resistant progenies (454064, 455035, 454053, 454058) and six susceptible progenies (454066, 454071, 455072, 455082, 454045, 454077)] were evenly divided among four trays (six plants per genotype per tray), with replicate plants planted in a row. A separated tray with six plants per genotype was prepared for non-inoculated control. After 4 weeks, at least five leaves were present on each plant and the youngest internode was tagged.

Spores from *D. ampelina* isolate Nita001 were produced according to Travadon et al. (2013) from an isolate originally collected in June 2015 from leaf spots on ‘Cabernet Sauvignon’ in Winchester, Virginia. The spore suspension was adjusted with sterile water to 1 × 10^6^ conidia ml^−1^ and was sprayed until runoff on the leaves and stems of plants using an atomizer (Mondi Mist & Spray Deluxe Tank Sprayer, Hydroframs, USA). Non-inoculated plants were sprayed in the same way, but with sterile water instead. Infection was encouraged by covering each tray with a dome (XL High Dome Propagator, Garland Products, England), maintaining greenhouse conditions at 20 °C and continuous light for 24 h. After this 24-h wetting period, domes were removed from each tray.

Disease severity on the internodes was assessed 30 days post-inoculation by estimating the percentage of the area covered by lesions on the four internodes below the internode tagged at inoculation, using a modified Horsfall–Barratt scale (Barratt and Horsfall 1945) with 12 levels (1: 0–5%; 2: 5–10%; 3: 10–20%; 4: 20–30%; 5: 30–40%; 6: 40–50%; 7: 50–60%; 8: 60–70%; 9: 70–80%; 10: 80–90%; 11: 90–100%; 12: 100%). Differences between genotypes were determined by ANOVA, as described in “Statistics”.

### Differential expression (DE) analysis in *V*. *cinerea* B9 after inoculation with *D*. *ampelina*

Six 1-year-old, 1 m tall *V. cinerea* B9 plants were acclimated in a lighted mist chamber (25 °C) 2 days before inoculation. Leaves were spray inoculated with a Preval handheld paint sprayer (Preval, IL, USA) using either a *D. ampelina* suspension isolated from field-infected vines as described, or sterile water (mock).

One leaf sample for each inoculation treatment was collected from three replicate vines (biological replicates) both before (T0, 3rd leaf) and 48 h post-inoculation (hpi) (T2, 4th leaf) (three replicates for each of two collection times for each of two inoculations conditions = 12 samples). Tissues were immediately stored in liquid nitrogen and transferred to the laboratory for RNA extraction. Total RNA was extracted using a Spectrum Plant Total RNA kit (Sigma-Aldrich, USA), after grinding frozen tissue to a fine powder with mortar and pestle. Barcoded, strand-specific, mRNA multiplexed libraries were prepared as previously described (Zhong et al. 2011). Each library was single-end (100 bp) sequenced on a HiSeq 2000 (Illumina Inc., USA) at the Genomics Facility of the Institute of Biotechnology at Cornell University.

RNA-Seq reads were processed with the Fastx toolkit for demultiplexing, barcode trimming and quality filtering (Pearson et al. 1997). Cutadapt was used to remove all residual adapter sequences (Martin 2011). Differential expression analysis of normalized FPKM (fragments per kilobase of exon per million fragments mapped) expression values was executed following standard protocols (Haas et al. 2013), with the following experiment-specific details. First, the RSEM software (Li and Dewey 2011) was used to align the quality reads to the *V. vinifera* PN40024 reference transcriptome (Grimplet et al. 2012). The trimmed mean of M-values (TMM) normalization method was executed in R to generate FPKM values for each transcript (Dillies et al. 2013).

After calculation of normalized expression values for each sample, DE genes after inoculation [false-discovery rate (FDR) ≤ 0.001] were determined for each inoculation treatment using the edgeR software (Robinson et al. 2010). The set of exclusive DE genes in samples inoculated with *D. ampelina* was obtained by subtracting genes that were DE after both pathogen and mock inoculation. These inoculated-exclusive DE genes were input for pathway enrichment analysis as previously described (Osier 2016). The experiment described in the above section is referred to as a DE study in the following sections.

### Genotyping and construction of genetic maps

Genotyping and genetic map construction for these families have been previously described (Hyma et al. 2015). Briefly, DNA was extracted from one young leaf per vine using the DNeasy^®^ 96 Plant Kit (Qiagen). Genotyping-by-sequencing (GBS) libraries (Elshire et al. 2011) were constructed at 384-plex and sequenced with an Illumina HiSeq 2000 DNA sequencer (single-end, 100 bp read length). SNP calling was performed according to the TASSEL 3.0 GBS pipeline (Glaubitz et al. 2014) using the *V. vinifera* PN40024 reference genome version 12X.0 (Adam-Blondon et al. 2011; Jaillon et al. 2007). SNP names indicate SNP position on the reference genome coded as S(chromosome)_(position in bp). GBS genotype information was used to identify vines derived from self-pollination or cross-contamination, which were removed from the family dataset. SNP filtering and parental genetic map construction (Table 1) utilized the de novo HetMappS pipeline, using pseudo-testcross markers only (Hyma et al. 2015).

**Table 1:** Total genetic map distance and number of SNPs for female and male maps of three *Vitis* F_1_ families

Family (# individuals)	Genetic distance (cM)		Number of SNPs	
	Female map	Male map	Female map	Male map
‘Horizon’ × Illinois 547-1 (366)	1286	1314	4316	5560
‘Horizon’ × *V. cinerea* B9 (162)	1347	1125	3118	1956
‘Chardonnay’ × *V. cinerea* B9 (148)	1275	1293	2394	2177

Genetic maps were created using the HetMappS de novo pipeline and curated with R/qtl

Additionally, for a subset of 94 DNA samples of progeny and parents of the ‘Horizon’ × *V. cinerea* B9 family, the following SSR markers located near the resistance loci were genotyped: VVIB22 (Merdinoglu et al. 2005), VrZAG62 (Sefc et al. 1999), VVMD7 (Bowers et al. 1996) and SC8_0040_088 (Jaillon et al. 2007). PCR reactions were performed with 6 µl of QIAGEN Multiplex PCR Plus Kit (Qiagen, Germany), 1 µl of primer mix (0.5 µM each) and 5 µl of each DNA sample diluted 1:10. PCR amplification was performed with 30 cycles of 95 °C for 30 s, 57 °C for 90 s and 72 °C for 90 s, followed by 68 °C for 30 min. Fragment sizes were determined relative to a LIZ 500 Size Standard using an ABI 3730xl (Applied Biosystems, USA) at the Genomics Facility of the Institute of Biotechnology at Cornell University. Allele calls were generated using GeneMarker V 2.4.0 (SoftGenetics, USA).

### QTL analysis

QTL were localized using the R/qtl package (Broman et al. 2003) implemented in the statistical software R (R Core Team 2014) as described previously (Barba et al. 2014). Multipoint probabilities were calculated using *calc.genoprob* with step = 1 and default parameters. Initial QTL positions were determined with the *scanone* function using a normal model, Haley–Knott regression and default parameters. LOD significance scores were determined by permutation tests (1000). Initial QTL positions were used to define QTL with the *makeqtl* function; significance of model terms was tested with *fitqtl* command and positions were refined with *refineqtl*. The *addqtl* command was used to test if another QTL was needed. A 1.5 LOD supported interval was determined using the *lodint* function, and QTL effects were calculated as the difference in the mean phenotype value of individuals within each genotype class at the marker or pseudomarker (a position between markers) with the highest LOD score, using the *effectplot* function in R/qtl.

### Expression QTL (eQTL) analysis

A subset of 12 cane-resistant (scores 0 or 1) and 12 cane-susceptible (score 2 or 3) progeny from ‘Chardonnay’ × *V. cinerea* B9 were selected to maximize the number of progeny with recombination events around the *Rda*1 resistance locus. On August 29, 2013, three shoots on each field-grown vine was spray inoculated using a Preval handheld paint sprayer (Preval, IL, USA) immediately before sunset and enclosed in a moistened plastic bag to maintain surface wetness. The next morning (at 14 hpi), inoculated stem internode between the second and third unfurled leaf was collected, immediately stored in liquid nitrogen and transferred to the laboratory for RNA extraction.

Strand-specific, mRNA multiplexed libraries and RNA-Seq reads were processed as described above. EdgeR was used to determine normalized expression values as FPKM (Trapnell et al. 2010) and to determine DE transcripts between the resistant and susceptible samples (12 samples each) with a false-discovery rate (FDR) significance threshold of FDR ≤ 0.05, after Benjamini–Hochberg multiple comparison corrections. This experiment is referred to as the eQTL study in the following sections.

### Statistics

Analysis of variance (ANOVA) was used to determine the percentage of internode lesions on the California greenhouse experiment, using a factorial model for fixed effects plant genotype and experiment. For this, % internode lesions were converted to the mid-point of the percent range for each scale value (e.g., 45% for a score of “6”). ANOVA was performed using the MIXED procedure in SAS, with Kenward–Roger as the denominator degrees of freedom method (Littell et al. 1996). Homogeneity of variance across treatments was confirmed according to (Box et al. 1978). For significant effects (*p* < 0.05), differences among means were assessed based on the overlap of their 95% confidence intervals, and means without overlapping intervals were considered significantly different (Westfall et al. 1999).

Correlation between ratings of disease severity on dormant canes in NY field and mean % internode lesions in California greenhouse was determined by the CORR procedure in SAS, based on the Spearman rank-order correlation (non-parametric measure of association, based on the ranks of the data values).

Linkage between SSR and GBS SNP markers was determined by a *χ*^2^ test of independence using the Chi square test function implemented in the stat package of R (R Core Team 2014) over a subset of 66 individuals.

Multiple comparison corrections of *p* values were performed with the Benjamini–Hochberg procedure implemented in the R multtest package (Pollard et al. 2004).

## Results

### Field symptoms and isolation of *Diaporthe ampelina*

**Fig. 2:**
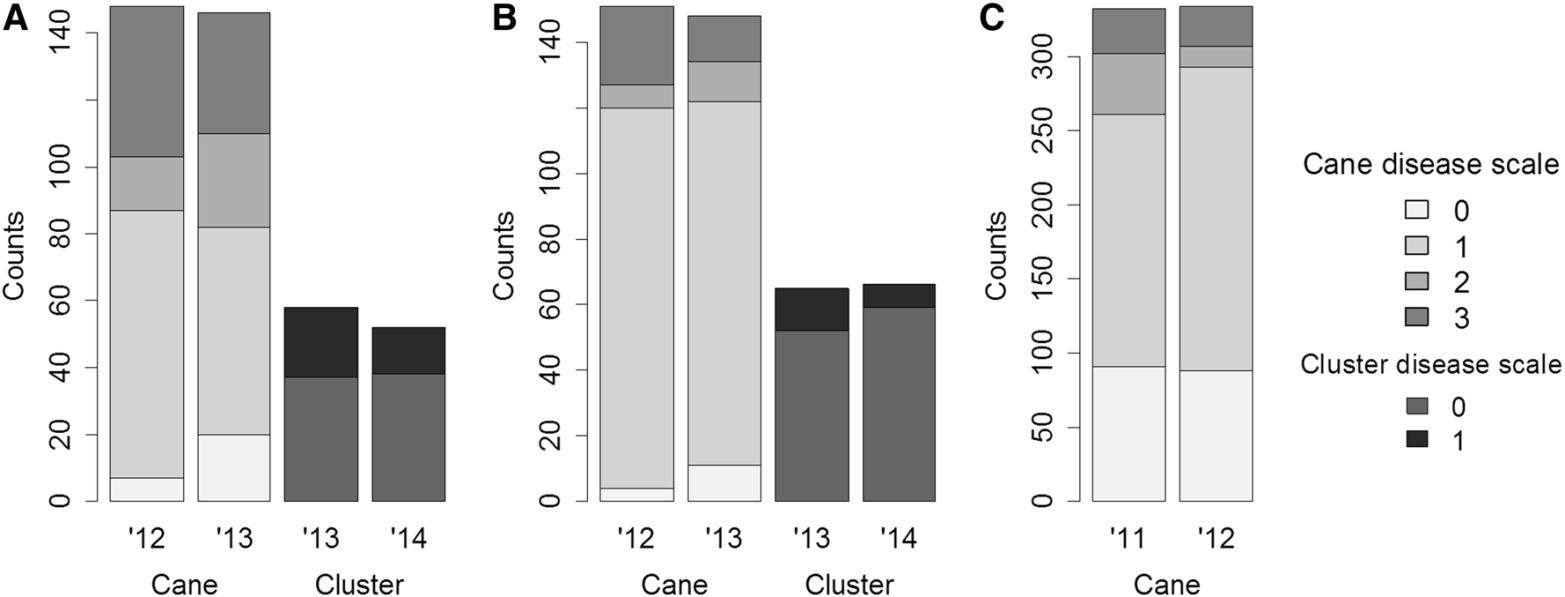
Segregation of dormant cane symptoms and cluster symptoms in three F_1_ families. **a** ‘Chardonnay’ × *V. cinerea* B9, **b** ‘Horizon’ × *V. cinerea* B9 and **c** ‘Horizon’ × Illinois 547-1. Disease severity on canes was measured for 2 years using the following scale: (0) no Phomopsis symptoms observed; (1) light infection, small number of discrete lesions; (2) moderate infection, lesions coalescing, widespread; and (3) severe infection, lesions blackened, internode tissue corky and misshapen. On clusters, symptoms such as black superficial spots, shriveled berries and dry rachis were scored as present (1) or absent (0). Across all years, average cane severity was 0.75, 0.88, 0.75 and 1 for ‘Chardonnay’, *V. cinerea* B9, ‘Horizon’ and Illinois 547-1, respectively and 0 for the cluster-bearing parents ‘Chardonnay’ and ‘Horizon’

In the field, lesions on dormant canes varied from absent (asymptomatic) to widespread with black, corky wood and canes that were visibly stunted (Fig. 1a). All parental genotypes showed few to no symptoms, having either a score of 0 (for one vine of ‘Chardonnay’, one vine of *V. cinerea* B9 and two vines of ‘Horizon’), or a score of 1 (three vines of ‘Chardonnay’, seven vines of *V. cinerea* B9, six vines of ‘Horizon’ and four vines of Illinois 547-1 displaying a small number of discrete cane lesions). While no parental plants showed scores of 2 or 3, these extremely susceptible phenotypes were observed for a proportion of progeny in all three F_1_ families (Fig. 2).

Often, vines with symptoms on the canes also developed fruit symptoms. On immature clusters, black spots appeared on the berry surface and lesions on the rachis were also observed (Fig. 1c, d). After veraison, rachis lesions became dry and blackened, and berries became shriveled or split (Fig. 1c). Cluster symptoms were absent from female parents and were not possible to observe with the dioecious male parents *V. cinerea* B9 and Illinois 547-1. Among progeny, cane symptom scores correlated with the presence of cluster symptoms, with Pearson’s *r* of 0.92 and 0.76 in 2012–2013 and 2013–2014, respectively (Fig. 1d). The typical leaf spot symptom was rare among all families. Samples from symptomatic dormant canes incubated in humid conditions developed pycnidia that exude conidia (cirrhi), typical of *D. ampelina*. Conidia from these samples were successfully cultured on PDA plates, producing colonies with typical growth rings and cream colored cirrhi of pycnidia (Fig. 1b). Isolation of fungi was not successful from symptomatic berries.

### Confirmation of phenotypes after *D. ampelina* inoculation

**Fig. 3:**
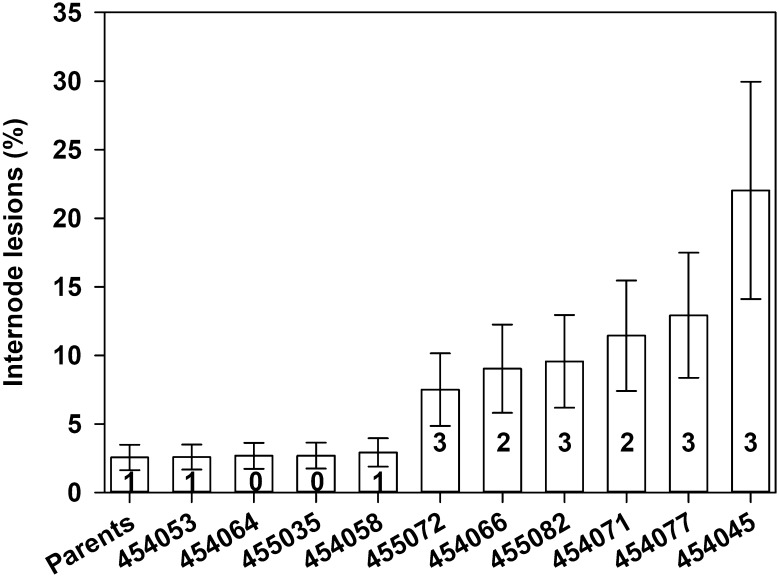
Internode lesions (%) on the green stems of ten progenies from the F_1_ family of ‘Chardonnay’ × *V. cinerea* B9, after inoculation with *D. ampelina* in the greenhouse. The proportion of stem surface, spanning the four assessed internodes, covered by lesions was visually estimated at 30 d post-inoculation. ‘Parents’ are pooled values for Chardonnay and *V. cinerea* B9. Numbers at the base of each column are field ratings of disease severity on canes (on a scale of 0–3, Fig. 1). Each column is the mean of three observations, averaged across three replicate experiments (24 plants per genotype per experiment). Error bars are 95% confidence limits. Columns with overlapping error bars are not significantly different (*p* < 0.05; Tukey’s test)

A subset of six susceptible and four resistant progenies from the ‘Chardonnay’ × *V. cinerea* B9 cross were inoculated in the greenhouse to confirm that genotypes with susceptible phenotypes on dormant canes in NY field also expressed susceptible phenotypes (more typical stem and internode symptoms of Phomopsis cane and leaf spot) in an independent experiment (California). Our findings in the greenhouse were consistent with field observations. ANOVA detected significant differences in % internode lesions among genotypes (*p* < 0.0001). The two most susceptible genotypes in the field, 454077 and 454045, also had the highest levels of internode lesions in the greenhouse (Fig. 3). Similarly, the most resistant genotypes, which had field ratings of 0 or 1 (454053, 455035, 454058), were not significantly different from the parents, both of which had field ratings of 1. Non-inoculated plants developed no lesions, which suggests that symptoms were not due to remnant inoculum from the field, but from a different isolate of *D. ampelina* applied. The Spearman rank-order correlation between dormant cane field scores and the ranking of accessions based on percentage of symptomatic area on green stems was *R*^2^ = 0.78 with *p* value of 0.004. Consistent with field observations, leaf spots were rare in the greenhouse, even among genotypes with susceptible stems.

### Transcriptome response of *V*. *cinerea* B9 to inoculation with *D*. *ampelina* (DE study)

To characterize the defense response of the resistant parent *V. cinerea* B9, we contrasted the expression of genes in *V. cinerea* B9 before (T0) and 48 h after (T2) inoculation with either *D. ampelina* or sterile water (mock). The mean number of sequencing reads obtained for this study was 10.3 million per replicate (supplementary Figure S1) or 30.9 million per treatment.

**Fig. 4:**
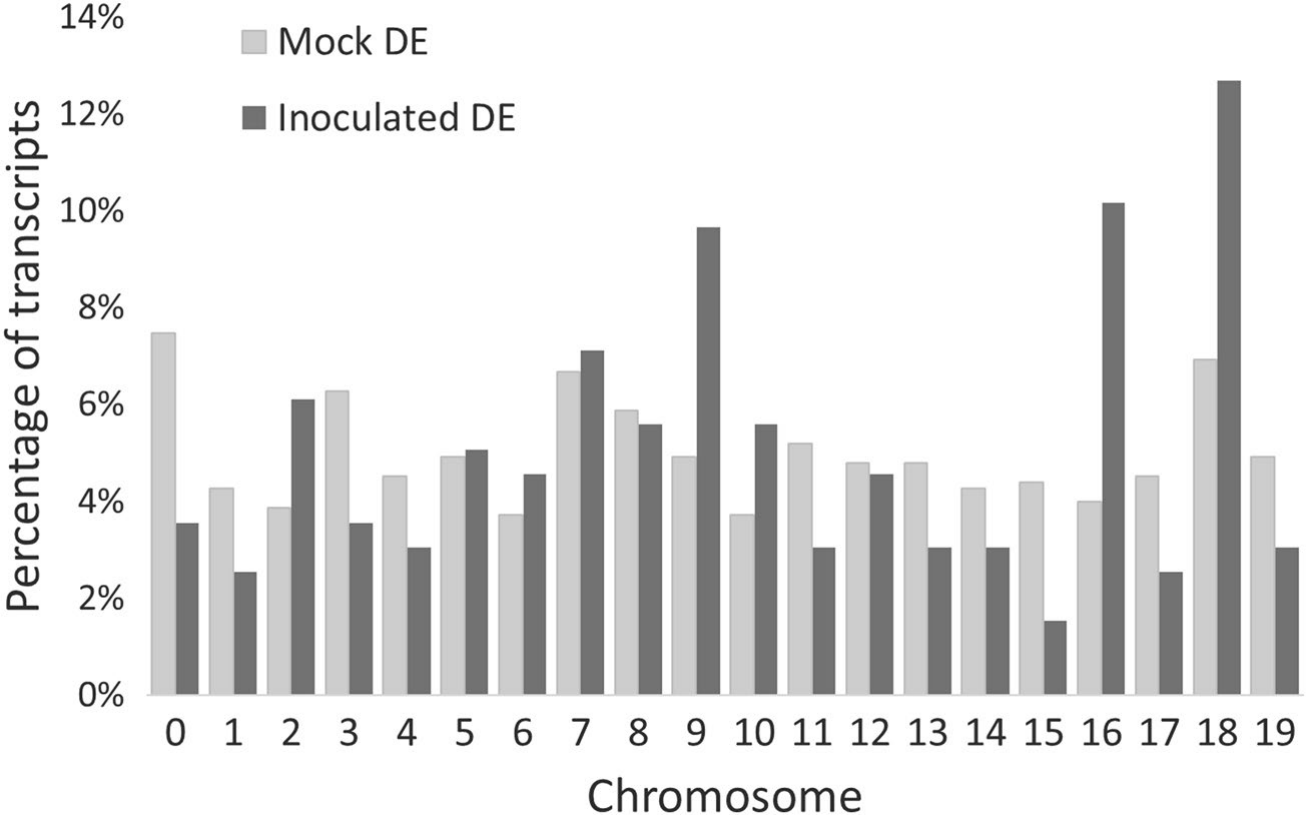
Chromosomal distribution of differentially expressed (DE) genes of *V. cinerea* B9 after inoculation with sterile water (mock DE, *n* = 751) or *D. ampelina* (inoculated DE, *n* = 290). For both treatments, genes with differential expression values between T0 (before inoculation) and T2 (48 h post-inoculation) were determined at FDR ≤ 0.001

In inoculated *V. cinerea* B9, the 197 DE genes (T2 vs T0 at FDR ≤ 0.001) were unevenly distributed across 19 chromosomes (1.5–12.7% per chromosome), with inoculation enriching DE of genes on chromosomes 2, 9, 10, 16 and 18 (Fig. 4). Notably, genes on chromosome 15 showed a 2.9-fold repression of DE over time in treated samples, from 4.4% in mock inoculated to 1.5% in inoculated (Fig. 4). A greater number of genes (754) were DE in mock-inoculated vines, but had a less uneven distribution across 19 chromosomes (3.7–7.5% per chromosome).

There were 122 shared DE genes between mock and *D. ampelina* inoculated samples. Inoculated-exclusive DE genes were strongly enriched in pathways associated with ethylene signaling and phenylpropanoid biosynthesis and significantly enriched in pathways associated with anthocyanin biosynthesis and nitrogen metabolism (Supplementary Table S2). Out of the 75 inoculated-exclusive DE genes, 56 genes (74.6%) were down-regulated 48 h after inoculation (Table 2), including several ethylene-responsive transcription factors and auxin-related proteins. Among others, pathogenesis proteins, peroxidases, stilbene synthase, tropinone reductase, dirigent protein and the cytochrome P450 hydroxylase CYP86A1 were up-regulated at 48 h after inoculation.

**Table 2:** *Vitis cinerea* B9 transcripts with *Diaporthe ampelina* inoculation-exclusive differential expression (DE) genes at 2 days post-inoculation, using false-discovery rate (FDR) ≤ 0.001

GeneID	Functional annotation	FDR	logFC
VIT_03s0063g00460	Ethylene-responsive transcription factor ERF109	5.0 × 10^−10^	− 8.01
VIT_07s0005g05910	Auxin-binding protein ABP19	5.2 × 10^−4^	− 6.30
VIT_11s0016g00660	DREB sub A-5 of ERF/AP2 transcription factor	2.7 × 10^−10^	− 5.34
VIT_16s0013g01060	Ethylene-responsive transcription factor ERF105	1.2 × 10^−4^	− 4.29
VIT_16s0013g01030	Ethylene-responsive transcription factor ERF105	3.4 × 10^−6^	− 3.63
VIT_09s0002g02030	Pyruvoyl-dependent arginine decarboxylase	7.9 × 10^−4^	− 3.60
VIT_09s0002g08060	2-Hydroxyacid dehydrogenases, D-isomer specific	8.2 × 10^−7^	− 3.56
VIT_16s0013g00950	Ethylene-responsive transcription factor ERF105	1.9 × 10^−7^	− 3.53
VIT_06s0009g03670	F-box family protein	1.0 × 10^−5^	− 3.45
VIT_08s0007g08520	Unknown protein	9.6 × 10^−14^	− 3.31
VIT_03s0180g00210	Myb domain protein R1	2.5 × 10^−5^	− 3.29
VIT_14s0066g02350	Galactinol synthase	9.6 × 10^−8^	− 3.26
VIT_01s0127g00700	Unknown protein	1.1 × 10^−15^	− 3.24
VIT_07s0005g01140	Unknown protein	6.3 × 10^−4^	− 3.21
VIT_13s0064g01110	No hit	5.3 × 10^−7^	− 3.17
VIT_16s0013g01050	Ethylene-responsive transcription factor ERF105	1.2 × 10^−7^	− 3.15
VIT_06s0009g01620	Harpin-induced protein	3.4 × 10^−6^	− 3.11
VIT_16s0013g00990	Ethylene-responsive transcription factor ERF105	3.3 × 10^−7^	− 3.10
VIT_06s0080g01090	CCR4-NOT transcription complex subunit 7/8	8.4 × 10^−11^	− 3.10
VIT_11s0016g01810	Unknown protein	1.8 × 10^−13^	− 3.10
VIT_18s0001g07320	2-Oxoglutarate/malate carrier protein, Mitochondrial	2.4 × 10^−9^	− 3.08
VIT_12s0134g00240	Avr9/Cf-9 rapidly elicited protein 20	1.3 × 10^−8^	− 2.98
VIT_14s0081g00520	ERF12	8.8 × 10^−5^	− 2.93
VIT_06s0004g04180	Zinc finger (C2H2 type) protein (ZAT11)	6.2 × 10^−4^	− 2.82
VIT_18s0001g09230	Salt-tolerance zinc finger	2.3 × 10^−5^	− 2.79
VIT_16s0013g00970	Ethylene-responsive element-binding factor 5	3.5 × 10^−7^	− 2.72
VIT_02s0025g02490	Unknown protein	2.0 × 10^−4^	− 2.70
VIT_16s0013g00980	Ethylene-responsive transcription factor ERF105	5.3 × 10^−4^	− 2.67
VIT_19s0093g00550	9-Cis-epoxycarotenoid dioxygenase 2	1.4 × 10^−4^	− 2.59
VIT_09s0054g01410	Beta-amyrin synthase	7.7 × 10^−9^	− 2.59
VIT_17s0000g01630	Calmodulin CML37	4.7 × 10^−4^	− 2.59
VIT_12s0028g03270	Ethylene-responsive transcription factor 9	1.5 × 10^−5^	− 2.54
VIT_18s0001g11170	Myb domain protein 73	9.2 × 10^−6^	− 2.54
VIT_12s0134g00170	No hit	2.5 × 10^−5^	− 2.53
VIT_16s0013g01000	Ethylene-responsive transcription factor ERF105	8.2 × 10^−4^	− 2.51
VIT_02s0012g02820	Geraniol 10-hydroxylase	2.1 × 10^−4^	− 2.46
VIT_07s0255g00020	OBF-binding protein 1	2.8 × 10^−4^	− 2.44
VIT_08s0105g00190	U-box domain-containing protein	1.5 × 10^−5^	− 2.43
VIT_19s0014g02240	Ethylene-responsive element-binding factor 4	6.7 × 10^−9^	− 2.43
VIT_18s0122g00300	Unknown protein	1.2 × 10^−5^	− 2.42
VIT_05s0077g01970	Zinc finger (C3HC4-type ring finger)	3.6 × 10^−9^	− 2.39
VIT_09s0002g08030	Arogenate dehydrogenase isoform 2	4.5 × 10^−5^	− 2.34
VIT_01s0011g04550	Unknown protein	1.1 × 10^−6^	− 2.32
VIT_18s0001g06560	No hit	8.1 × 10^−9^	− 2.30
VIT_05s0020g04570	CBL-interacting protein kinase 7 (CIPK7)	2.7 × 10^−5^	− 2.20
VIT_03s0038g02140	Auxin transporter protein 2	5.4 × 10^−4^	− 2.19
VIT_18s0122g00980	Glucan endo-1,3-beta-glucosidase 7 precursor	2.5 × 10^−4^	− 2.16
VIT_17s0000g09270	MATE efflux family protein	1.5 × 10^−5^	− 2.16
VIT_00s0267g00030	Unknown	2.9 × 10^−4^	− 2.15
VIT_18s0166g00190	U-box domain-containing protein	4.1 × 10^−4^	− 2.15
VIT_00s0218g00140	Anthocyanidine rhamnosyl-transferase	4.8 × 10^−4^	− 2.14
VIT_18s0001g09910	l-Asparaginase	3.7 × 10^−7^	− 2.14
VIT_02s0012g02810	CYP76C4	1.2 × 10^−4^	− 2.13
VIT_15s0048g02070	BON2-associated protein (BAP2)	3.4 × 10^−4^	− 2.09
VIT_16s0050g01580	UDP-glucose: anthocyanidin 5,3-*O*-glucosyltransferase	8.2 × 10^−4^	− 2.06
VIT_03s0063g00830	Carboxyesterase 5 CXE5	1.7 × 10^−4^	− 2.04
VIT_15s0107g00550	Tetratricopeptide repeat domain male sterility MS5	1.2 × 10^−5^	2.14
VIT_16s0100g00930	Stilbene synthase 2	1.5 × 10^−4^	2.61
VIT_00s0229g00190	Inositol 2-dehydrogenase like protein	1.4 × 10^−6^	2.66
VIT_16s0039g01300	Phenylalanine ammonia-lyase (*Vitis vinifera*)	4.3 × 10^−4^	2.73
VIT_16s0100g00810	Stilbene synthase (*Vitis vinifera*)	3.3 × 10^−7^	2.82
VIT_05s0077g01560	Pathogenesis protein 10.3 (*Vitis quinquangularis*)	3.8 × 10^−9^	3.12
VIT_16s0100g00900	Stilbene synthase (*Vitis pseudoreticulata*)	3.5 × 10^−7^	3.22
VIT_16s0100g00860	Chalcone synthase	1.4 × 10^−7^	3.48
VIT_16s0100g01030	Stilbene synthase (*Vitis quinquangularis*)	1.2 × 10^−5^	3.54
VIT_05s0077g01550	Pathogenesis protein 10.3 (*Vitis quinquangularis*)	8.9 × 10^−5^	3.56
VIT_18s0001g06850	Peroxidase GvPx2b class III	1.1 × 10^−7^	3.67
VIT_05s0077g01530	Pathogenesis protein 10 (*Vitis vinifera*)	1.7 × 10^−8^	3.71
VIT_05s0077g01570	Pathogenesis protein 10 (*Vitis vinifera*)	3.2 × 10^−12^	3.80
VIT_16s0100g01150	Stilbene synthase (*Vitis vinifera*)	1.4 × 10^−6^	4.13
VIT_08s0007g00920	Tropinone reductase	8.8 × 10^−5^	4.13
VIT_04s0069g00730	Glutamate receptor protein	7.3 × 10^−4^	4.18
VIT_07s0031g01680	CYP86A1	5.1 × 10^−4^	4.34
VIT_06s0004g01020	Dirigent protein	4.3 × 10^−5^	4.62
VIT_16s0100g00940	Stilbene synthase 3 (*Vitis* sp. cv. ‘Norton’)	2.9 × 10^−4^	4.94

Fifty-six ###genes with negative log2 fold change (logFC) were down-regulated after inoculation (logFC < − 2), and nineteen genes with positive logFC were up-regulated after inoculation (logFC > 2)

### QTL analysis

The high-density genetic maps used for this analysis were derived from genotyping by sequencing using the pseudo-testcross approach. Since the 12X.0 version of the PN40024 reference genome was used, markers have both physical and genetic positions (Hyma et al. 2015).

Two major loci located on chromosomes 15 and 7 from the *V. cinerea* B9 and ‘Horizon’ parents, respectively, showed dominant effects and significantly predicted both the severity and incidence of cane and cluster symptoms, respectively, for all years and families tested (Table 3). Here, we refer to these *V. cinerea* B9 and ‘Horizon’ loci as *Rda*1 and *Rda*2, respectively. For all crosses used in this study, vines with either the *Rda*1 or *Rda*2 resistance allele had either no symptoms or small, discrete lesions (scores 0 or 1), while vines with both susceptible alleles showed moderate to severe symptoms (scores 2–3). On the Illinois 547-1 map, two other minor QTL were significant only in the 2011 evaluation of dormant canes and explained much less of the phenotypic variance (3.2 and 3.5%) than *Rda*1 or *Rda*2 (28.4 and 24.8%) (Table 3). No QTL was identified from ‘Chardonnay’.

**Table 3:** QTL mapping statistics

Family	Parent	Chr^a^	Phenotype	Year	Peak Marker^a^ (cM)	Left Marker^a^ (cM)	Right Marker^a^ (cM)	LOD	PVE^c^ (%)
‘Chardonnay’ × *V. cinerea* B9	*V. cinerea* B9	15	Cane	2012	S15_19560016 (62.2)	S15_19299979 (61.4)	S15_19591520 (63.7)	51.4	79.8
				2013	S15_19560016 (62.2)	S15_19299979 (61.4)	S15_19591520 (63.7)	44.2	75.2
			Cluster	2013	S15_19591520 (63.7)	S15_18780806 (57.1)	S15_20031941 (66.8)*	16.5	73.0
				2014	S15_19560016 (62.2)	S15_18780806 (57.1)	S15_20031941 (66.8)*	10.5	60.7
‘Horizon’ × *V. cinerea* B9	‘Horizon’	7	Cane	2012	S7_2768585 (15.2)	S7_1087848 (10.0)	S7_3855744 (19.1)	50.4	41.1
				2013	S7_3127568 (15.5)	S7_1087848 (10.0)	S7_3855744 (19.1)	29.1	48.0
			Cluster	2013	S7_3127568 (15.5)	S7_1087848 (10.0)	S7_4952429 (24.7)	30.6	22.8
				2014	S7_1860119 (13.9)	S7_1087848 (10.0)	S7_4952429 (24.7)	25.1	11.2
	*V. cinerea* B9	15	Cane	2012	S15_19591538 (51.4)	S15_19560016 (50.6)	S15_19637245 (53.1)*	56.1	51.0
				2013	S15_19591538 (51.4)	S15_19560016 (50.6)	S15_19637245 (53.1)*	32.2	56.3
			Cluster	2013	S15_19591538 (51.4)	S15_19560016 (50.6)	S15_19637245 (53.1)*	30.1	20.0
				2014	S15_19637245 (53.1)	S15_19560016 (50.6)	S15_19637245 (53.1)*	26.5	20.4
‘Horizon’ × Illinois 547-1	‘Horizon’	7	Cane	2011	S7_2000903 (6.5)	S7_1459378 (4.5)	S7_2409624 (7.7)	30.3	24.8
				2012	S7_1912889 (5.6)	S7_1459378 (4.5)	S7_2409624 (7.7)	58.4	45.5
	Illinois 547-1	1	Cane	2011	S1_3046182 (11.3)	S1_1170106 (3.8)	S1_4279265 (14.5)	3.6	3.2
		2		2011	S2_5852870 (34.5)	S2_2340804 (12.0)	S2_7231845 (40.5)	4.7	3.4
		15		2011	S15_19300044 (49.2)	S15_19053446 (46.1)	S15_19591538 (54.7)	34.2	28.4
				2012	S15_19300044 (49.2)	S15_19300044 (49.2)	S15_19591538 (54.7)	58.6	46.1

Loci associated with Phomopsis cane and berry symptoms were identified by multiple QTL mapping on parental maps for three families

^a^Chromosome (Chr) and marker positions correspond to the physical location in the 12X.0 PN40024 *Vitis vinifera* reference genome. Markers are reported in the format S(chromosome)_(position in bp). Left and right markers correspond to the closest marker to the borders of a 1.5 LOD interval. An asterisk (*) indicates the last marker of the map

^b^LOD threshold was determined by permutation test (1000), at *α* = 0.05, and ranged from 2.90 to 3.14

^c^PVE refers to the percentage of variance explained by the locus

According to the physical position of flanking markers, the smallest supported interval for the *Rda*1 locus is located between 19.3 and 19.6 Mbp of chromosome 15 (with higher LOD of 51.4 and 58.6, Table 3), and the smallest supported interval for the *Rda*2 locus is located between 1.5 and 2.4 Mbp of chromosome 7 for the result with higher LOD (Table 3, LOD 58.4). There are 39 annotated genes within the 300 kb supported interval for the *Rda*1 locus, which codes for five NB-LRR proteins (Grimplet et al. 2012) that are potentially associated with plant–pathogen interactions.

### SSR markers associated with resistance locus

Based on a subset of 94 individuals, three SSR markers, VVIB22 (Merdinoglu et al. 2005), VrZAG62 (This et al. 2004), and VVMD7 (Bowers et al. 1996) located on chromosome 7, near the *Rda*2 locus, were confirmed to be linked to *Rda*2 (Table 4). SC8_0040_088 (Jaillon et al. 2007) was the only SSR marker near *Rda*1 in the PN40024 reference (18.9 Mbp), but was homozygous in the resistant parent *V. cinerea* B9 (358 bp) and thus non-informative in the progeny.

**Table 4:** SSR allele sizes in linkage with the *Rda*2 locus

SSR marker	Physical location (Mbp)	‘Horizon’		*V. cinerea* B9	
		Allele size (bp)	*p* value	Allele size (bp)	*p* value
VVIB22	3.10	**157**/139	1.8 × 10^−12^	144/160	0.068
VrZAG62	1.78	**180**/202	1.8 × 10^−12^	174/188	0.650
VVMD7	1.17	**237**/235	1.2 × 10^−11^	231/231	na

Alleles associated with resistance to Phomopsis cane lesions from ‘Horizon’ are indicated in bold. Linkage was determined by *χ*^2^ test over a subset of 66 progeny from ‘Horizon’ × *Vitis cinerea* B9

### Association of the *Rda*1 locus with gene expression (eQTL study)

**Fig. 5:**
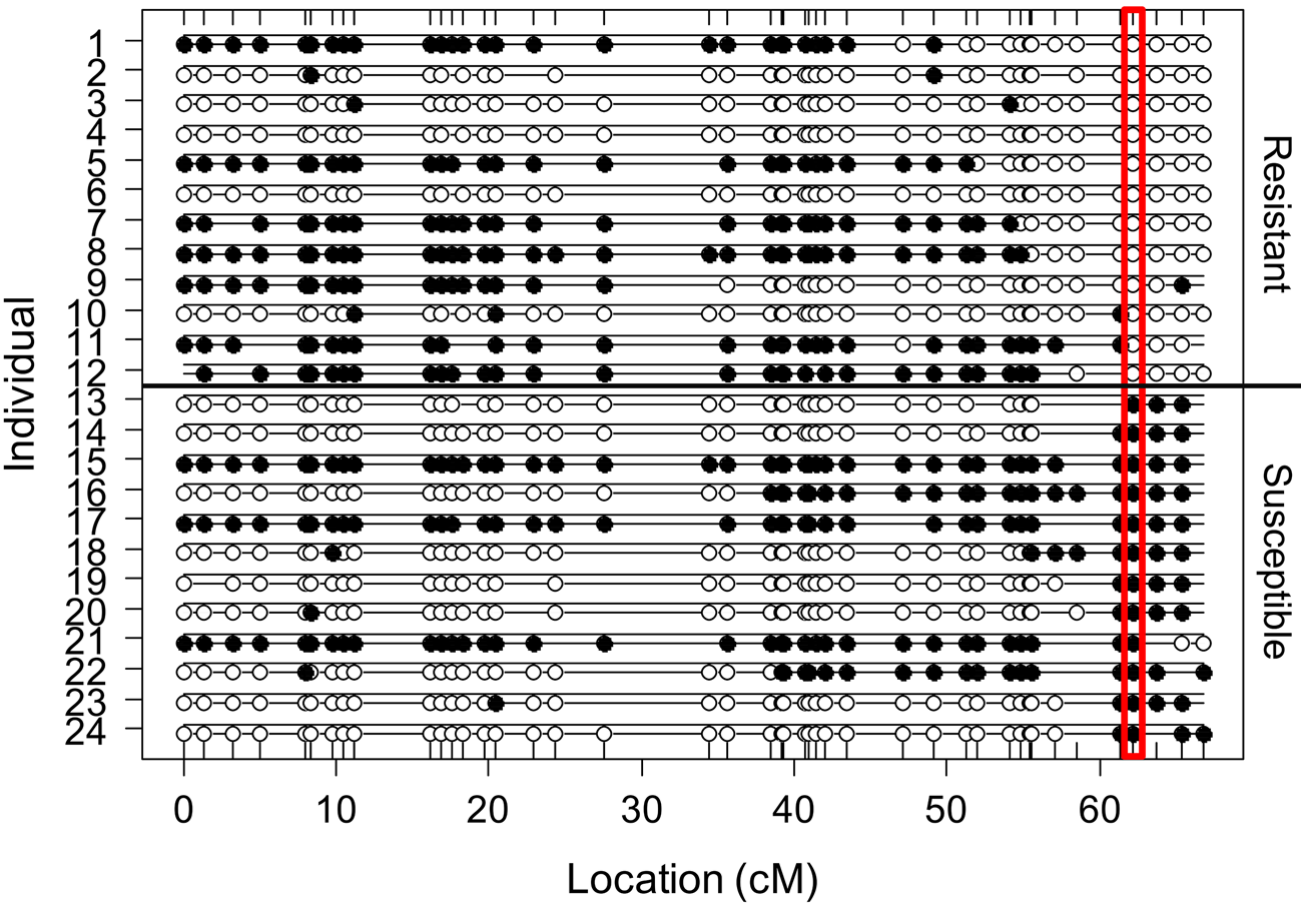
Genotypes on chromosome 15 of the *Vitis cinerea* B9 map for individuals selected for RNA-Seq. Resistant (upper) and susceptible (lower) progeny showed genotype segregation at the *Rda*1 locus. The marker with highest LOD score is indicated. White and black dots indicate the allelic states AAxBA and AAxAB, respectively

We used an eQTL approach to further investigate the association between expression of candidate genes and the resistance locus. For this, 12 resistant and 12 susceptible vines from the ‘Chardonnay’ × *V*. *cinerea* B9 progeny were sampled, 14 of which exhibited recombination within 15 cM of the *Rda*1 locus (Fig. 5). The total number of quality reads was 32.01 million per treatment (± *Rda*1 allele), with a sample mean and median of 2.67 million reads and 2.20 million reads per progeny, respectively (Supplementary Figure S1).

We found 16 DE genes between resistant and susceptible progeny 14 h after inoculation, at FDR ≤ 0.05, including three NB-LRR genes on chromosome 15 (Supplementary Table S3). Expression was significantly predicted by alleles at the *Rda*1 locus for 6 of these 16 genes: a mannitol dehydrogenase gene and the three aforementioned NB-LRRs on chromosome 15 were up-regulated in susceptible vines, while a cytochrome P450 monooxygenase gene (CYP78A3p) and an auxin-responsive protein IAA17 gene were up-regulated in resistant vines (Table 5). The NB-LRRs VIT_15s0046g02730 and VIT_15s0046g02800 located at 19.45 and 19.53 Mbp, respectively, were the only two eQTL located within the *Rda*1 interval in the PN40024 12X.0 reference genome.

**Table 5:** Differential expression (DE) and expression QTL (eQTL) mapping statistics

Gene	Gene chr^a^	Gene position (bp)^a^	Gene functional annotation	logFC^b^	DE *p* value	eQTL LOD	eQTL LOD Thr^c^	eQTL PVE^d^	eQTL effect
VIT_15s0046g02730	15	19,454,696–19,457,671	PRF disease resistance protein	2–4	0.001	6.3	3.5	70.1	6.06
VIT_15s0021g00120	15	9,388,654–9,389,448	RPP13 recognition of *Peronospora parasitica* 13	25.3	6 × 10^−11^	5.1	3.6	62.7	19.9
VIT_15s0046g02800	15	19,528,135–19,530,195	PRF disease resistance protein	6.5	0.005	4.8	3.4	60.3	1.92
VIT_00s0346g00110	Un	24,788,096–24,791,922	Mannitol dehydrogenase	4.5	0.027	3.1	3.0	44.8	6.30
VIT_15s0048g02900	15	17,005,384–17,007,131	Cytochrome P450 monooxygenase CYP78A3p	− 3.4	0.029	3.1	2.6	45.0	− 5.90
VIT_09s0002g05160	9	4,853,689–4,862,025	Auxin-responsive protein IAA17	− 2.4	0.029	3.6	3.0	50.1	− 12.6

Six genes showed association between transcription levels and the *Rda*1 locus, located between 19.3 and 19.6 Mbp of chromosome 15

Genetic maps were used for multiple QTL mapping of transcription levels (FPKM) of differentially expressed genes on a subset of 24 progeny from ‘Chardonnay’ × *V. cinerea* B9

^a^Chr and gene positions correspond to the physical location in the 12X.0 PN40024 *Vitis vinifera* reference genome; chr Un corresponds to the unassembled pseudo chromosome

^b^logFC corresponds to the log2 fold change

^c^LOD Thr (threshold) was determined by permutation test (10,000) at *α* = 0.05

^d^PVE refers to the percentage of transcript variance explained by the *Rda*1 locus

## Discussion

This study started with observations of Phomopsis symptoms among segregating families in different environments and proceeded to genetic mapping of loci controlling the resistance phenotype. Within the *Rda*1 locus, we used transcriptome screening to narrow down candidates to two NB-LRR loci, providing the first clue for the molecular resistance mechanism for this pathogen.

First, we found complementary evidence from experiments in the field and greenhouse that there is genetic resistance to Phomopsis cane and leaf spot among plant genotypes representative of segregating families, based on observations of symptoms on different grapevine organs (canes, shoots, and clusters). Our findings of Phomopsis susceptibility in powdery mildew-resistant breeding materials makes it clear that cessation of fungicide use to minimize the latter could exacerbate the former. Understanding the genetics of resistance to Phomopsis cane and leaf spot on these organs could facilitate better strategies for its management in diverse environments, such as the east and west coasts of the USA, where the pathogens of both diseases are a problem.

Segregation ratios observed in the three hybrid families suggested the presence of one major dominant locus in ‘Chardonnay’ × *V. cinerea* B9 (1 resistant:1 susceptible) and at least two major dominant loci in ‘Horizon’ × *V. cinerea* B9 (3 resistant:1 susceptible) and ‘Horizon’ × Illinois 547-1 (*V. rupestris* B38 × *V. cinerea* B9). This observation was corroborated by QTL mapping, where the loci *Rda*1 and *Rda*2 were found in *V. cinerea* B9 and ‘Horizon’, respectively. Co-localization of loci obtained from cane and cluster phenotypes suggests that resistance in both tissues was due to the same loci. Therefore, we used the *Rda*1 and *Rda*2 designations for both phenotypes.

Both *Rda*1 and *Rda*2 loci showed major dominant effects that suggested qualitative resistance, providing protection against disease symptoms. As a consequence, the molecular markers provided for *Rda*2 can be used directly for marker-assisted selection of resistant vines at the seedling stage or for stacking Phomopsis resistance alleles along with resistance to major grapevine pathogens. For *Rda*1, readily assayed markers for MAS can be obtained from the GBS-SNPs provided in Table 3, as described previously (Yang et al. 2016).

Even though ‘Chardonnay’ disease severity was similar to that of *V. cinerea* B9 in both the greenhouse and field, and to ‘Horizon’ in the field, we were not able to identify resistance loci from ‘Chardonnay’. If ‘Chardonnay’ resistance is quantitative, our experiment may not have had enough statistical power to detect minor effect QTL. Among ‘Chardonnay’ × *V. cinerea* B9 F_1_ progeny, segregation of ‘Chardonnay’ loci can only be observed among progeny with *Rda*1 susceptible alleles, reducing the effective size of the population to fewer than 100 individuals. While ‘Chardonnay’ resistance could instead be recessively inherited, the rare presence of extremely susceptible phenotypes in related *V. vinifera* cultivars argues against that possibility.

The extremely susceptible phenotype observed in progeny that did not inherit a resistance allele was not typical of Phomopsis cane and leaf spot symptoms or fruit symptoms seen in commercial vineyards. This may be expected, since cultivated and bred grapes have been subjected to selection, which may have purged these extremely susceptible phenotypes. As an example, in cultivated grapevines, *D. ampelina* fruit infections initiated at the pedicel are typically latent during most of summer and symptoms do not appear until harvest, when berries rot and black pycnidia form (Wilcox et al. 2015). At our field site, symptoms were unusually severe, with black lesions appearing on the berries and rachises drying even before veraison. In some cases, berries did not even expand, but instead remained stunted and became necrotic. We did not recover *D. ampelina* from symptomatic clusters, as we did from the canes; nonetheless, the correlation between cluster and cane symptoms was evident. The cane symptoms observed in the field started out as lesions on the green stems; levels of cane symptoms in the field and internode lesions in the greenhouse on genotypes were positively correlated under both conditions.

At 48 hpi with *D. ampelina*, the resistant parent *V. cinerea* B9 was symptomless, with a transcriptome showing a complex profile with elements typical of immune responses mediated by NB-LRR and repression of antagonistic pathways. Up-regulated genes, such as pathogenesis-related proteins along with salicylic acid signaling genes, are consistent with defense responses to biotrophic pathogens. Other mechanisms of defense were also present, such as strengthening of physical defenses by the up-regulation of peroxidase class III, cytochrome P450 hydrolase CYP86A1 gene, involved in the biosynthesis of suberin (Hofer et al. 2008), or by up-regulation of a dirigent protein gene, involved in tissue lignification (Davin and Lewis 2000). Other up-regulated genes were associated with the production of defense-related secondary metabolites, such as stilbene synthases or tropinone reductase, related to alkaloid metabolism (Drager 2006). Down-regulation of genes involved in the ethylene signaling pathway as well as the auxin signaling pathway is required to activate the antagonistic salicylic acid (SA) pathway (Chang et al. 2015; Kazan and Manners 2009) and is also consistent with a biotrophic immune response. A possible future experiment could include validation of these expression differences by qRT-PCR.

High-density genetic maps, derived from sequencing small fragments and alignment to the *V. vinifera* PN40024 reference genome, allowed the immediate localization of flanking SNP markers in the physical map without the need for BAC libraries or further sequencing. For *Rda*1, the smallest supported interval of 300 kb between 19.3 and 19.6 Mbp on chromosome 15, determined in the result with higher LOD (Table 3, LOD 51.4 and 58.6), contains a cluster of NB-LRR genes (Grimplet et al. 2012). As described in other pathosystems, NB-LRR genes are candidates for qualitative resistance to a hemibiotrophic pathogen, such as *D. ampelina*. For *Rda*2, the smallest supported interval had less resolution, being located between 1.46 and 2.41 Mbp of chromosome 7 for the result with higher LOD (Table 3, LOD 58.4). This larger region of approximately 950 kb has 134 annotated genes (Grimplet et al. 2012) and no obvious candidate.

To further investigate the *Rda*1 locus, 24 full sibling progenies were used to delineate candidate resistance genes with an eQTL approach at 14 hpi. To increase the statistical power of this analysis, we followed some simple steps: first, we used the saturated genetic maps and the *Rda*1 locus position to identify vines with nearby recombination. These vines were sampled and used for construction of RNA-Seq libraries to provide better mapping resolution by maximizing the recombination events within and near the locus. Then, we focused the eQTL analysis by selecting those transcripts that were DE between vines with resistant and susceptible phenotypes, reducing the number of eQTL tests from 30,034 annotated PN40024 transcripts to only 16 DE transcripts (Supplementary Table S3). An eQTL scan for loci predicting expression of these 16 genes identified two candidate NB-LRR genes (VIT_15s0046g02730 and VIT_15s0046g02800) as differentially expressed and significantly associated to the *Rda*1 locus. We also identified one NB-LRR gene physically distant on chromosome 15 (according to the PN40024 reference) and three genes on other chromosomes regulated by the *Rda*1 locus early after *D. ampelina* inoculation: a mannitol dehydrogenase, the cytochrome P450 monooxygenase CYP78A3p and the auxin-responsive protein IAA17 (Table 5). While the eQTL hits located on *Rda*1 can point out two candidate R-genes for this locus, hits from other loci could be related to reactions triggered by ETI.

The elevated number of NB-LRR gene transcripts up-regulated in the susceptible progeny compared with the resistant progeny suggests that susceptibility and not resistance is mediated by the action of NB-LRR genes, which may facilitate the necrotrophic phase of the hemibiotrophic fungus. This is reminiscent of the wheat NB-LRR protein Tsn1, which confers sensitivity to ToxA from the necrotrophic fungi *Stagonospora nodorum* and *Pyrenophora tritici*-*repentis* (Faris et al. 2010). Alternatively, sequence divergence between the resistance allele and the reference transcriptome derived from *V. vinifera* ‘Pinot noir’ may have resulted in a misalignment of reads, resulting in lower FPKM values for the resistance allele. To elucidate this issue, a de novo transcriptome for the resistant parent was constructed using the DE study total reads, eQTL analysis was repeated and associated transcripts were annotated. We obtained the same eQTL as reported when using the reference genome; specifically, three of the four most significant DE genes were NB-LRR genes 80- to 410-fold up-regulated in susceptible versus resistant progeny (data not shown).

Another explanation is that the time point used was not representative of the plant response. For this, expression differences should be validated using qRT-PCR at several time points between inoculation and the appearance of symptoms on the susceptible vines. Moreover, a comparison of the molecular response between resistant and susceptible progenies to the biotrophic and necrotrophic phases of the pathogen would help to elucidate the hypothesis of susceptibility mediated by NB-LRR genes as stated above.

In RNA-Seq experiments, we used 14 hpi for field (eQTL experiment) and 48 hpi for growth chamber inoculations (DE experiment) to study the early response of the plant. As described, the beginning of *D. ampelina* life cycle suggests a biotrophic phase (Willison et al. 1965) that, in a successful interaction, develops lesions after 7 days, which are typically associated with its necrotrophic phase (Wilcox et al. 2015). This suggests that both experiments were conducted during the biotrophic phase of the pathogen, which is consistent with the absence of symptoms at the time of sample collection, and the plant transcriptome response activated toward a biotrophic response. In other pathosystems, such as the hemibiotrophic pathogen *Phytophthora infestans* in potato, 48 hpi also showed consistency between the absence of symptoms and a transcriptome response associated with a plant–biotroph pathogen interaction (Zuluaga et al. 2016).

The set of DE genes identified in the eQTL study of progeny vines differs from that in the DE study of the *V. cinerea* B9 parent. This is expected as consequences of distinct experimental designs. Differences in the *V. cinerea* B9 DE study reflected changes in expression between time points (0 and 48 hpi) for a single parental genotype. In contrast, differential expression in the eQTL study was among different genotypes (full siblings) at a single time point (14 hpi), obscuring whether genes were constitutively or dynamically differential. As the resistant *Rda*1 allele is dominant, observing the effect of the susceptible allele was only possible in the eQTL study, in which the two *Rda*1 alleles segregate, but not in the *V. cinerea* B9 DE study. Moreover, in the eQTL experiment, ‘Chardonnay’ alleles are also present, which can change the transcriptome profiles related to the *V. cinerea* B9 DE study. Regardless of these differences, both approaches suggest that NB-LRR gene-mediated host responses may be critical in determining the outcome of infection.

In summary, we report phenotypic, genetic and genomic information regarding the interaction between the pathogen *D. ampelina* and grapevines, including two novel resistance loci, *Rda*1 and *Rda*2 located on chromosome 15 of *V. cinerea* B9 and chromosome 7 of ‘Horizon’, respectively. In the case of *Rda*1, our results suggest that the *D. ampelina*—*V. cinerea* B9 interaction is mediated by the action of one or more NB-LRR genes with homology to VIT_15s0046g02730 and VIT_15s0046g02800, providing the first hints about the molecular basis of the Phomopsis resistance phenotype.

### **Author contribution statement**

BIR and LCD: conceived of and designed the experiments, provided populations and resources and reviewed the manuscript. JL: conducted process and statistical analysis of RNA-Seq data. RSL: provided first field assessment of symptoms and designed the evaluation scale, MO: performed statistical analysis of RNA-Seq. PB: conceived of and designed the experiments, collected phenotypic data, performed molecular biology and microbiology laboratory work, genetic analysis and statistical analysis, and wrote the paper. KB and RT: conceived and designed greenhouse inoculation assays, collected phenotypic data, performed statistical analyses and contributed to and reviewed the manuscript. WFW: performed field identification and isolation of *D. ampelina* and reviewed the manuscript.

## Electronic supplementary material

Below is the link to the electronic supplementary material.

Supplementary Figure S1: Distribution of number of quality reads per sample for differential expression (DE) and eQTL studies. (PDF 22 kb)

Supplementary material 2 (PDF 23 kb)

Supplementary material 3 (PDF 13 kb)
