# Supplementary material for: Two dominant loci determine resistance to Phomopsis cane lesions in F1 families of hybrid grapevines
Source: Theor Appl Genet. 2018 Feb 21;131(5):1173–89. doi: 10.1007/s00122-018-3070-1 (PMC5895676; doi:10.1007/s00122-018-3070-1)
Supplement: Supplementary file 1 — Supplementary Figure S1: Distribution of number of quality reads per sample for differential expression (DE) and eQTL studies. (PDF 22 kb) [file 122_2018_3070_MOESM1_ESM.pdf]

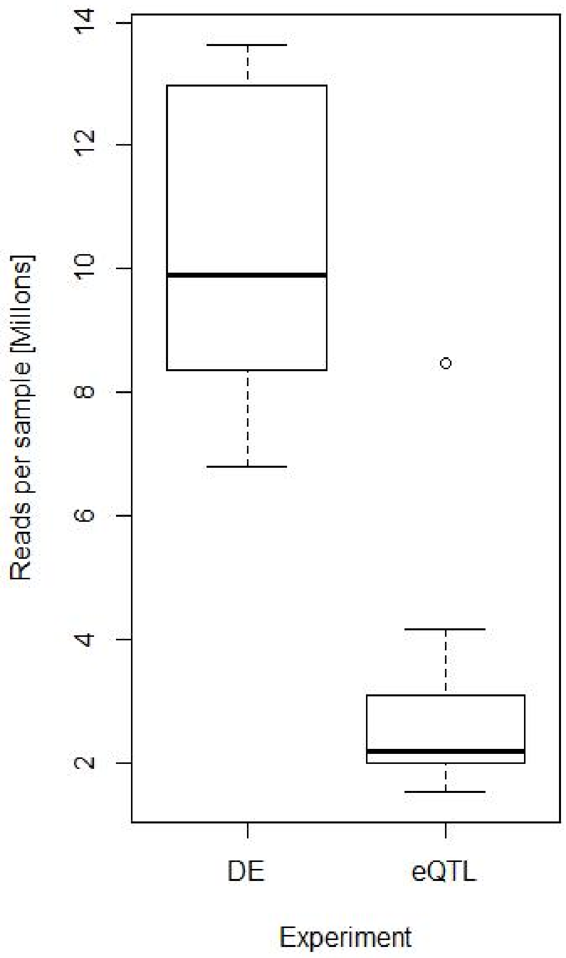

Supplementary Figure S1: Distribution of number of quality reads per sample for differential expression (DE) and eQTL studies. Libraries containing 12 and 24 barcoded samples were pooled for DE and eQTL, respectively. For each study, a RNA-Seq library was prepared according to (Zhong *et al.*, 2011) and single-end sequenced in separate lanes on a HiSeq2000 (Illumina Inc, USA).
