# Supplementary material for: Two dominant loci determine resistance to Phomopsis cane lesions in F1 families of hybrid grapevines
Source: Theor Appl Genet. 2018 Feb 21;131(5):1173–89. doi: 10.1007/s00122-018-3070-1 (PMC5895676; doi:10.1007/s00122-018-3070-1)
Supplement: Supplementary file 2 — Supplementary material 2 (PDF 23 kb) [file 122_2018_3070_MOESM2_ESM.pdf]

Supplementary Table S2: Pathway enrichment analysis for infection-exclusive differentially expressed (DE) genes from *Vitis cinerea* B9 inoculated with *Diaporthe ampelina* as described (Osier, 2016). Pathways are presented as assigned in VitisNet (Grimplet *et al.*, 2012).

Pathway		DE in pathway	not DE in pathway	DE not in pathway	not DE not in pathway	Fisher's Exact Test	permuted p-value
vv30008	Ethylene signaling	12	261	36	51,169	$2.2*10^{-16}$	0
vv60003	AP2 EREBP	13	145	35	51,285	$2.2*10^{-16}$	0
vv10940	Phenylpropanoid biosynthesis	7	236	41	51,194	$3.0*10^{-9}$	0
vv10942	Anthocyanin biosynthesis	2	70	46	51,360	0.0021	0.017
vv10910	Nitrogen metabolism	2	115	46	51,315	0.0054	0.042
vv11013	ABA biosynthesis	1	17	47	51,413	0.017	0.055
vv10220	Urea cycle and metabolism of amino groups	2	132	46	51,298	0.0070	0.066
vv60015	C2C2-DOF	1	24	47	51,406	0.023	0.077
vv60017	C2H2	2	136	46	51,294	0.0074	0.078
vv60044	MYB	2	177	46	51,253	0.012	0.105
vv10906	Carotenoid biosynthesis	1	48	47	51,382	0.045	0.130
vv10360	Phenylalanine metabolism	2	222	46	51,208	0.019	0.134
vv50004	Auxin transport	1	57	47	51,373	0.053	0.134
vv50123	Porters cat 18 to 29	2	223	46	51,207	0.019	0.136
vv10630	Glyoxylate and dicarboxylate metabolism	1	87	47	51,343	0.079	0.195
vv10562	Inositol phosphate metabolism	1	69	47	51,361	0.063	0.197
vv23018	RNA degradation	1	114	47	51,316	0.10	0.248
vv10251	Glutamate metabolism	1	96	47	51,334	0.087	0.261
vv10252	Alanine and aspartate metabolism	1	116	47	51,314	0.10	0.291
vv10680	Methane metabolism	1	132	47	51,298	0.12	0.328
vv10400	Phenylalanine tyrosine and tryptophan biosynthesis	1	158	47	51,272	0.14	0.357
vv34020	Calcium signaling pathway	1	151	47	51,279	0.13	0.369
vv10052	Galactose metabolism	1	175	47	51,255	0.15	0.375

Supplementary Table S2 (continuation): Pathway enrichment analysis for infection-exclusive differentially expressed (DE) genes from *Vitis cinerea* B9 inoculated with *Diaporthe ampelina* as described (Osier, 2016). Pathways are presented as assigned in VitisNet (Grimplet *et al.*, 2012).

Pathway		DE in pathway	not DE in pathway	DE not in pathway	not DE not in pathway	Fisher's Exact Test	permuted p-value
vv30001	ABA signaling	1	166	47	51,264	0.14	0.387
vv10350	Tyrosine metabolism	1	164	47	51,266	0.14	0.400
vv10941	Flavonoid biosynthesis	1	189	47	51,241	0.16	0.426
vv10900	Terpenoid biosynthesis	1	191	47	51,239	0.16	0.435
vv10902	Monoterpenoid biosynthesis	1	216	47	51,214	0.18	0.439
vv50125	Porters cat 66 to 94	1	229	47	51,201	0.19	0.477
vv60078	Other zf-C3HC4	1	276	47	51,154	0.23	0.541
vv30003	Auxin signaling	1	309	47	51,121	0.25	0.587
vv34626	Plant-pathogen interaction	1	359	47	51,071	0.29	0.631
vv10500	Starch and sucrose metabolism	1	372	47	51,058	0.29	0.662
vv50101	Channels and pores	1	449	47	50,981	0.34	0.734
