# Supplementary material for: Two dominant loci determine resistance to Phomopsis cane lesions in F1 families of hybrid grapevines
Source: Theor Appl Genet. 2018 Feb 21;131(5):1173–89. doi: 10.1007/s00122-018-3070-1 (PMC5895676; doi:10.1007/s00122-018-3070-1)
Supplement: Supplementary file 3 — Supplementary material 3 (PDF 13 kb) [file 122_2018_3070_MOESM3_ESM.pdf]

Supplementary Table S3: Differentially expressed (DE) genes between susceptible and resistant progeny segregating for *Rda*1, after multiple test correction by false discovery rate (FDR), α = 0.05. twelve transcripts were up-regulated on susceptible progenies (logFC > 0) and four transcripts were up-regulated on resistant progenies (logFC <0). Gene ID and functional annotation correspond to (Grimplet *et al.*, 2012). chr Un corresponds to *V. vinifera* PN40024 “random” chromosome. logFC correspond to

GeneID	Chr	Functional Annotation	FDR	logFC
VIT 15s0021g00120	15	RPP13 (recognition of *Peronospora parasitica* 13)	$5.6*10^{-11}$	4.60
VIT 15s0046g02730	15	R protein PRF disease resistance protein	0.001	1.29
VIT 15s0046g02800	15	R protein PRF disease resistance protein	0.005	2.69
VIT 08s0007g07670	8	NAC domain containing protein 47	0.006	5.90
VIT 08s0040g01500	8	High-affinity nitrate transporter 2.4	0.025	-4.13
VIT 00s0346g00110	Un	Mannitol dehydrogenase	0.027	2.20
VIT 18s0117g00550	18	Laccase	0.027	4.54
VIT 04s0008g04920	4	2-oxoglutarate-dependent dioxygenase	0.029	3.80
VIT 09s0002g05160	9	IAA17	0.029	-1.25
VIT 01s0011g03730	1	myb domain protein 62	0.029	3.24
VIT 12s0055g00510	12	NAC domain containing protein 104	0.029	2.12
VIT 05s0049g00600	5	No hit	0.029	2.85
VIT 15s0048g02900	15	CYP78A3p	0.029	-1.77
VIT 19s0014g01060	19	Sesquiterpene synthase	0.047	-4.85
VIT 17s0000g08450	17	Carbonic anhydrase chloroplast	0.047	3.93
VIT 05s0020g03710	5	GCN5 N-acetyltransferase (GNAT)	0.047	2.35
